# Anther Culture-Derived Haploids of *Citrus aurantium* L. (Sour Orange) and Genetic Verification of Haploid-Derived Regenerated Plants

**DOI:** 10.3390/plants11223022

**Published:** 2022-11-09

**Authors:** Seong Beom Jin, Min Ju Kim, Cheol Woo Choi, Suk Man Park, Su Hyun Yun

**Affiliations:** Citrus Research Institute, National Institute of Horticultural & Herbal Science, RDA, Jeju 63613, Korea

**Keywords:** aneuploidy, callus culture, chromosome analysis, ploidy, simple sequence repeat, somatic embryo

## Abstract

Citrus plants are important fruit tree species; however, the breeding of high-quality varieties of citrus species is a time-consuming process. Using haploid-derived plants from anther culture may reduce the time required for obtaining purebred lines. This study aimed to genetically verify whether anther culture-derived sour orange (*Citrus aurantium* L.) plants developed from somatic embryos or haploid tissues. Sour orange anthers were cultured in N6 and MS media to induce calli and somatic embryos. N6 liquid medium supplemented with 1 mg·L^−1^ gibberellic acid and 200 µM spermidine resulted in a 10% increase in callus and embryo induction rates. Regenerated plants were validated using simple sequence repeat markers. Out of the 109 regenerated plants, ploidy analysis identified 99 diploids, two haploids, and eight putative aneuploids; out of the 99 diploid plants, 33 were haploid-derived homozygous diploids. The chromosomal analysis confirmed most plants as diploids, whereas some were identified as aneuploids (19–21 chromosomes). Furthermore, phylogenetic analysis confirmed that the resultant homozygous or heterozygous plants were haploid-derived. This is the first report of haploid-derived homozygous diploid and aneuploid sour orange plants obtained through anther culture. Moreover, the anther cultivation technique described herein can be applied to other citrus varieties.

## 1. Introduction

Citrus plants belong to the family Rutaceae, which includes some of the most economically important and globally widespread fruit tree crops [1,2,3,4]. In terms of the cultivated area and production of domestic fruit trees, citrus fruits rank third (after apples and pears) and are in high supply and demand [5]. Although sour orange (*Citrus aurantium* L.) propagates through seeds, it is mainly cultivated using rootstocks [6,7]. Recent studies have shown that this species has various pharmacological properties, including anti-cancer, anti-anxiety, anti-obesity, antibacterial, antioxidant, insecticidal, and antidiabetic effects [8,9]. However, sour orange is highly susceptible to infection by citrus tristeza virus [6,10]. Furthermore, sour oranges produce numerous seeds, which is a major disadvantage in terms of its market value [11]. Despite these drawbacks, it has high utility as it can be effectively used as a tissue culture model [12].

Generating superior lines of citrus cultivars using conventional breeding methods, such as crossing, is time-consuming and labor-intensive owing to their high heterozygosity, long juvenility, self-incompatibility, and polyploidy [1,13,14,15,16,17]. Furthermore, breeding and selection of purebred lines are time-consuming processes [18]. However, compared to conventional breeding, anther culture facilitates the generation of haploid plants in a shorter period of time. This allows the production of completely homozygous lines from heterozygous parents in a single generation [15,19]. This technique also permits the immediate confirmation of recessive traits and phenotypes (for example, morphological features such as dwarfism) since haploid plants are generally smaller and exhibit significantly less vigor than their parents [18,20,21].

Haploid plants cannot undergo normal meiosis, which hinders generational progress [18,19]. In contrast, double haploids (homozygous diploids) obtained through natural or artificial treatments undergo normal meiosis, thus allowing generational progress [18]. Consequently, homozygous individuals obtained from haploids have important applications in crop breeding, such as mutagenesis, transformation, genetic analysis, and gene sequencing [15,22,23]. Therefore, anther culture has a high potential for plant breeding and crop improvement [24,25], and has been studied in a wide range of plant species [26,27]. To date, citrus plants obtained using anther culture include *Poncirus trifoliata* (L.) Raf. (trifoliate orange), *Citrus clementina* Hort. ex Tan. (clementine), *Citrus sinensis* (L.) Osbeck (sweet orange), *Citrus sinensis* (L.) Osbeck (Valencia sweet orange), *Citrus limon* (L.) Burm. f. (lemon), and *Clausena excavata* [1,28,29,30,31,32]. However, systematic breeding of citrus varieties using a combination of different pure lineages is insufficient for obtaining more pure-lineage varieties. Therefore, it is necessary to develop a suitable anther culture technique that can be used to obtain more pure-lineage varieties in large numbers.

Aneuploidy is a type of chromosomal anomaly that involves an increase or decrease in the number of chromosomes [33] and is caused by the non-separation of chromosomes during meiosis and mitosis [34,35,36]. While aneuploidy causes serious disorders in animals [35,37], in plants, the known consequences include myriad phenotypic and structural changes, such as growth retardation [38,39,40,41]. Although aneuploidy occurs spontaneously in some plants [42], it has also been reported to occur through crossbreeding in many crops, such as tobacco, corn, guava, pear, apple, and citrus [43,44,45,46]. Moreover, anther culture has been used to produce aneuploid tobacco plants [47]. Among haploid-derived plants obtained from some maize lines and anthurium plants, the incidence of aneuploidy is reportedly up to 15% [48,49]. Aneuploid plants have also been generated in citrus cultivars through anther culture [30,34,50,51]. The frequent occurrence of aneuploid individuals—including haploids and diploids—in plants obtained through anther culture [49] requires further investigation.

In this study, an optimum medium for anther culture of sour orange was developed. This medium was used to culture citrus embryos to determine the efficiency of anther culture of sour orange plants. Furthermore, the ploidy status of haploid-derived plants was verified and the associated genetic changes were investigated. Additionally, chromosomal analysis was used to verify the presence of homodiploid and aneuploid plants, and phylogenetic analysis was used to confirm whether homozygous or heterozygous plants were haploid-derived.

## 2. Results

### 2.1. Plant Regeneration System

**Figure 1:**
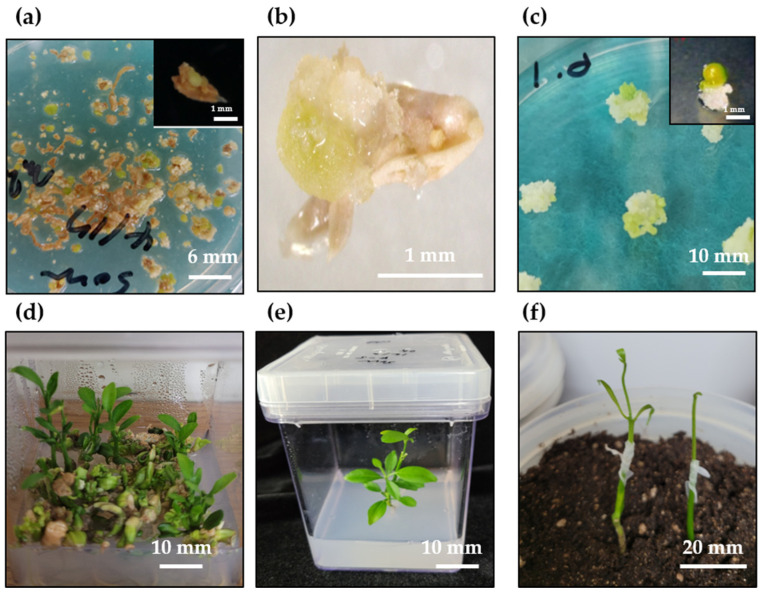
Plant regeneration from anther culture of *Citrus aurantium* L. (sour orange). (**a**) Callus and embryo induction on N6 liquid medium supplemented with GA_3_ (1 mg·L^−1^) and spermidine; (**b**) formation of callus and direct somatic embryo caused by bursting anther; (**c**) proliferation of induced callus cells and induction of somatic embryo; (**d**) plant regeneration; (**e**) in vitro grafting; (**f**) in vivo grafting.

The anthers swelled within 1–2 weeks; a few callus cells and embryos formed in 3–4 weeks (Figure 1a,b). When N6 liquid medium was supplemented with 1 mg·L^−1^ GA_3_ and 200 µM spermidine was added to the anther culture medium, the rates of callus induction and embryo formation increased by approximately 10%; the original callus induction rate was 25% and that of embryo formation was approximately 2% without the addition of the liquid medium (Table S1). The obtained callus cells were transferred to a medium for somatic embryo induction to induce embryo formation (Figure 1c). After 6 weeks of culture, the induced somatic embryos were transferred to a plant regeneration medium, and normal plants were obtained at a rate of approximately 50–60% after 4–6 weeks of culture (Figure 1d). Normal plants with roots and shoots were transferred to Murashige and Skoog (MS) medium without hormones. The remaining abnormal embryos were transferred to MS medium containing 500 mg·L^−1^ malt extract, 50 g·L^−1^ sucrose, and 0.5 mg·L^−1^ GA_3_, which induced continuous plant growth from non-regenerated calli and embryos at a rate of approximately 70–80%. Normal plants were rapidly induced to proliferate and grow through in vitro and in vivo grafting (Figure 1e,f). Ultimately, more than 1000 regenerated plants were obtained using the anther culture method.

### 2.2. Genetic Verification

**Figure 2:**
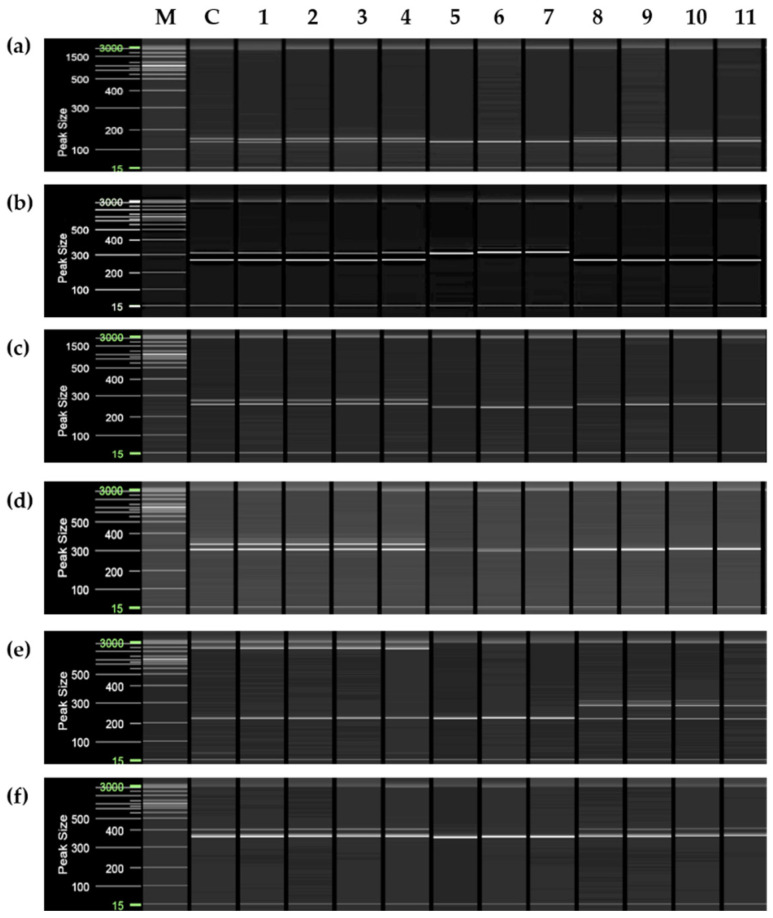
Molecular analysis of simple sequence repeat (SSR) marker in control and regenerated plants obtained from the anther culture. Polymerase chain reaction was performed using the following primers: (**a**) BM-CiSSR-P1; (**b**) BM-CiSSR-43; (**c**) BM-CiSSR-226; (**d**) BM-CiSSR-246; (**e**) BM-CiSSR-254; and (**f**) BM-CiSSR-260. M, molecular marker (20 and 100 bp DNA ladder); C, control plant; lanes 2–11, regenerated plants obtained using anther culture.

To confirm whether the plants obtained through anther culture were haploid-derived, eight simple sequence repeat (SSR) markers (CiSSR-P1, -P2, -43, -226, -246, -253, -254, and -260) out of eight combinations of heterozygous SSR markers were used. Out of >1000 plants obtained through anther culture, 271 were randomly selected for analysis using P1 and P2 markers (Table S2). Of the 271 plants, 11 were randomly selected for analysis using all eight SSR markers. Amplification using the SSR marker CiSSR-P1 showed four plants (lanes 1–4; Figure 2a) with similar amplification patterns (double band) as that in the control (donor plant). In contrast to the control, three of the seven putative haploid-derived plants (lanes 5–7) showed a single amplification product. The remaining four plants (lanes 8–11) did not show a single amplification product, but their amplification patterns differed from those of the control.

The SSR marker CiSSR-43 showed two amplification bands, similar to that shown by CiSSR-P1 in the control (Figure 2b). Putative heterozygous plants (lanes 1–4) also showed the same amplification pattern of bands as the control plants (Figure 2c). However, three of the seven putative haploid-derived plants (lanes 5–7) exhibited the same pattern as the upper amplification band of the control. Four other plants (lanes 8–11) had the same pattern as the lower amplification band of the control (Figure 2b). In four plants (lanes 1–4; Figure 2c,d) regenerated from anther culture, the SSR markers CiSSR-226 and CiSSR-246 showed the same amplification pattern (double band as in the control. Seven regenerated plants (lanes 5–11) that were putatively haploid-derived showed a single amplification product (Figure 2c,d). In the case of the CiSSR-246 marker, putative haploid plants (lanes 5–7) showed a pattern of double amplification bands, which differed from those of the control.

The SSR markers CiSSR-254 and CiSSR-260 showed two amplification bands, which was similar to the pattern exhibited by the CiSSR-P1 marker in the control (Figure 2e,f). Three of the seven putative haploid-derived plants (lanes 5–7) showed a single band pattern. However, the other four plants (lanes 8–11) had a different amplification pattern (double amplification bands) from that of the control (Figure 2e,f).

**Figure 3:**
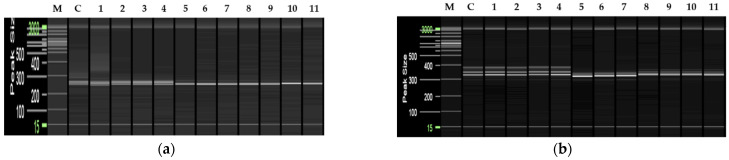
Simple sequence repeat (SSR) molecular analysis of the control and regenerated plants obtained using anther culture. Polymerase chain reaction analysis using the primers (**a**) BM-CiSSR-P2; (**b**) BM-CiSSR-253. M, molecular marker (20 and 100 bp DNA ladder); C, control plant; lanes 2–11, regenerated plants obtained through anther culture.

Unlike CiSSR-P1, the SSR markers CiSSR-P2 (Figure 3a) and CiSSR-253 (Figure 3b) showed three amplification bands in the control (Figure 3). Putative heterozygous plants (lanes 1–4) showed the same amplification band pattern as those of the control (Figure 3). However, putative haploid-derived plants (lanes 5–11) showed a single band pattern (Figure 3). The abovementioned results (Table S2) indicate that approximately 84% (228 plants among a total of 271) were heterozygous plants derived from the anther wall, and approximately 13% (35 plants among a total of 271) were haploid-derived homozygous; a few plants (approximately 3%; eight plants out of 271) were presumed as aneuploid plants (Table S2).

### 2.3. Polyploidy Verification and Morphological Selection

**Figure 4:**
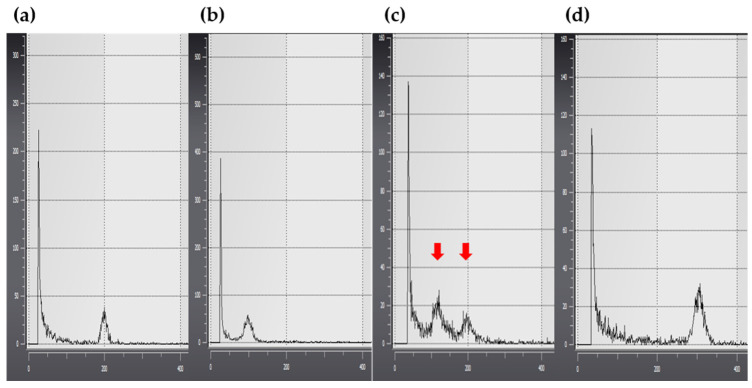
Ploidy analysis using flow cytometry. (**a**) Donor plant used as the control; (**b**) Haploid plants obtained through anther culture; (**c**) Mixed haploids and diploids obtained through anther culture; (**d**) Putative aneuploid plants obtained through anther culture. The red arrows indicate patterns associated with polyploidy.

Polyploidy was investigated in 109 plants obtained through anther culture. These included 41 out of 43 (two specimens were lost due to contamination) genetically selected plants and 68 genetically validated plants (Figure 4 and Table S3).

Verification of ploidy in the 109 plants identified about 91% of plants as diploids (66 of the 109 plants were heterozygous diploids; 33 were haploid-derived homozygous diploids; Figure 4a); about 2% of the plants were identified as haploids (two plants; Figure 4b,c), and 7% were identified as putative aneuploids (eight plants; Figure 4d and Table S3).

**Figure 5:**
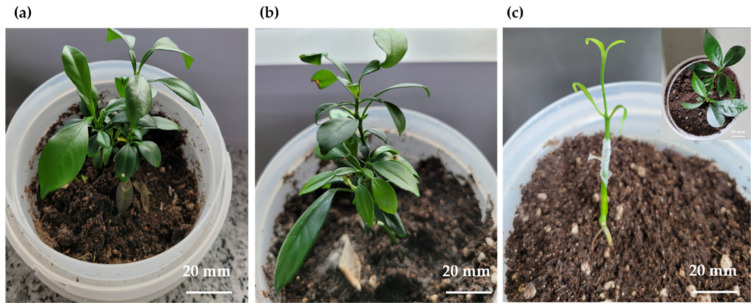
Morphological characteristics of plants produced using anther culture. Plants obtained through anther culture after approximately 2 months of in vivo grafting. (**a**), hetero-diploid plant (control); (**b**,**c**), putative haploid-derived regenerated plants.

Although most of the haploid-derived diploid plants (Figure 5b) were morphologically similar to the control plants (Figure 5a), morphological differences were observed in some of the plants (Figure 5c) in terms of leaf width, size, and growth rate. Moreover, the roots of putative haploid-derived plants appeared as thin threads; either the shoots failed to form, or the roots grew without shoot elongation (sample lost due to contamination).

### 2.4. Chromosomal Analysis

**Figure 6:**
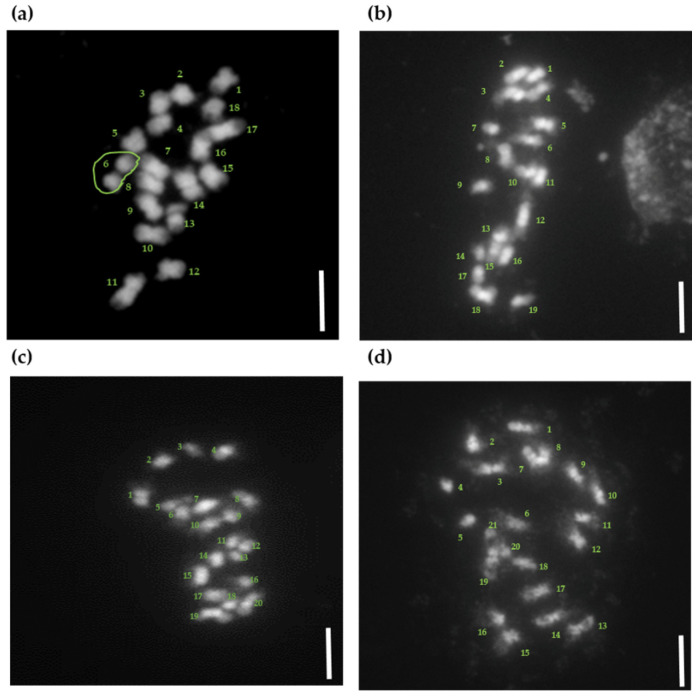
Chromosomal analysis of plants obtained through sour orange anther culture. (**a**) Diploid plants (2n:18); (**b**) Aneuploid plants (2n:19); (**c**) Aneuploid plants (2n:20); (**d**) Aneuploid plants (2n:21). Numbers in green indicate chromosome numbers; scale bar = 5 µm.

The number of chromosomes was analyzed in the root tips of 30 haploid-derived plants identified using SSR markers (Table S2). Most plants (22 of 30 plants) were identified as diploids with 18 chromosomes (Figure 6a and Table S4). In contrast, some plants (8 of 30 plants) that appeared to be either diploids or triploids were identified as putative aneuploids with 19–21 chromosomes (Figure 6b–d and Table S4).

### 2.5. Phylogenetic Analysis

**Figure 7:**
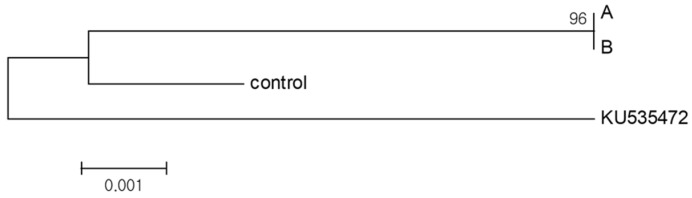
Maximum likelihood phylogenetic tree of anther culture-derived regenerated plants based on nucleotide sequences in the internal transcribed spacer (ITS; ITS1 + 5.8S rDNA + ITS2) region. A and B: regenerated plants obtained through anther culture; control: donor and heterozygous plants; Ku535472: accession number of “sour orange” registered in the GenBank.

**Figure 8:**
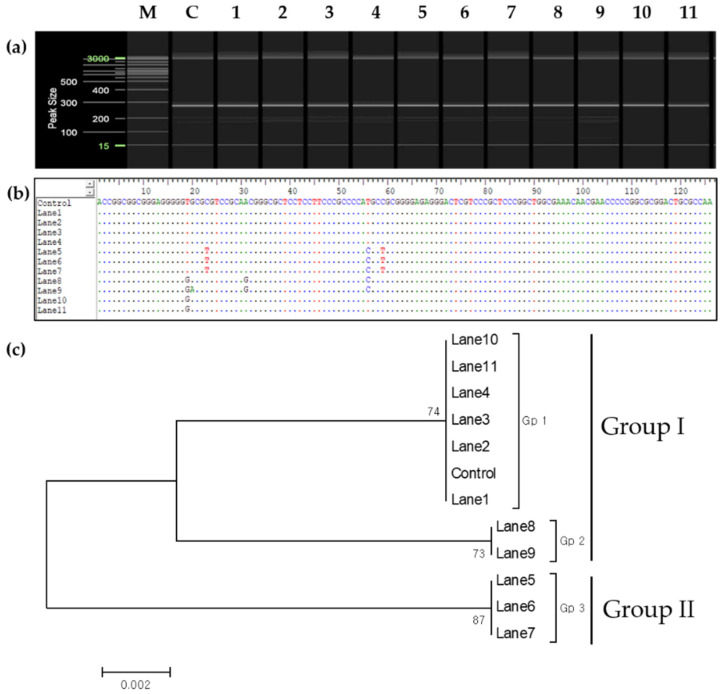
Maximum likelihood phylogenetic tree of regenerated anther-derived plants based on sequences in the internal transcribed spacer (ITS) 1 region. (**a**) Amplification patterns of the ITS1 region. (**b**) Alignment of the nucleotide sequence in the ITS1 region. Lanes 1–11: plants obtained through anther culture; control: leaf from the donor plant. (**c**) Phylogenetic analysis. M, molecular marker (20 and 100 bp DNA ladder); C, control plant; GP, subgroup.

The internal transcribed spacer (ITS) chromosomal regions were analyzed to determine the genetic relationship between the donor and regenerated plantlets and to establish whether the anther culture-derived plants were somatically mutated or haploid-derived. The results indicated a genetic difference in the chromosomal ITS region between plants identified using SSR markers and the control plants (Figure 7). Subsequently, genetic relationships between these plants were analyzed using nucleotide sequences in the ITS1 region to determine the zygosity of cultivars identified using SSR markers (Figure 8a,b). Phylogenetic analysis allowed the clustering of cultivars into two large groups and three small groups: Group 1 included Gp1 (Figure 8c: control, lanes 1–4, and lanes 10 and 11) and Gp2 (Figure 8c: lanes 8 and 9), and Group 2 included Gp3 (Figure 8c: lanes 5–7).

## 3. Discussion

This study investigated embryo formation and callus induction in sour oranges using anther culture to obtain haploid and double-haploid plants. The production of haploid- derived plants through anther culture is more challenging in citrus cultivars than in herbaceous species [1] because *Citrus* plants are characterized by long juvenility [14]. Successful production of haploid plants using anther culture has primarily been reported in grain species (especially Cruciferae) and some Solanaceae species [52,53,54,55]. However, recent studies have reported successful embryo induction and plant regeneration through anther culture in *Citrus* species [1,16,17,56]. In addition, the generation of haploid (homozygous diploid) and triploid plants has also been reported [16,17,34,57]. Among *Citrus* species, sour orange varieties are widely used as rootstocks for industrial use and tissue culture. The rootstocks also serve as useful models for anther culture [6,27]. However, there are no previous reports on the generation of putative haploid or aneuploid plants, as most anther-derived plants are reportedly diploids [12].

Previous studies on anther culture in sour orange cultivars [1,12,27] have shown that N6 medium is more effective than MS medium (both supplemented with growth hormones) for embryo and callus formation, although no significant differences have been reported between the two media. Both gibberellic acid (GA_3_) and spermidine are indicated in callus induction or stimulate somatic embryogenesis [57,58]. In this study, the N6 liquid medium supplemented with spermidine and GA_3_, when added to the solid medium during culturing of sour orange anthers, caused swelling of the anthers and exerted a greater effect on embryo and callus formation (Table S1). A previous study also showed that the addition of liquid medium to a solid medium [59] proved effective for anther culture in several plant species, including barley [60], wheat [61], tobacco [62], and black pepper [63]; similar results were obtained in this study (Figure 1 and Table S1). However, the composition of plant hormones used for anther culture in this study differed from those used in previous studies [1,12,27,31]. Satisfactory results were obtained when a combination of plant growth regulators similar to that described by Chiancone et al. [57] was used (Figure 1 and Table S1).

Genetic verification of plants obtained through anther culture was performed for 271 plants using eight combinations of heterozygote-specific SSR markers (Figure 2 and Figure 3). Therefore, eight SSR markers (CiSSR-P1, -P2, -43, -226, -246, -253, -254, and -260) were confirmed to indicate whether haploid plants were present. The results confirmed the existence of haploid-derived and heterozygous plants. PCR amplification products that were not found in the control were also identified. Through the verification of ploidy, most plants were confirmed as diploids. Some plants were also determined to be putative diploids or triploids; however, based on chromosomal analysis, it was confirmed that an aneuploid plant had 19–21 chromosomes instead of 27. Previous studies have reported that approximately 80% of anther culture-derived clementine plants were triploid when the anther culture was subjected to spermidine treatment [30,57]. However, this pattern was not observed in the present study, which could be attributed to differences between citrus varieties.

Aneuploid plants were obtained with two types of leaf morphologies; leaves of aneuploid plants were either narrower or wider than those of their diploid counterparts. This may be attributed to spermidine treatment, which stimulates embryogenesis during anther culture [57]. To confirm whether these individuals were formed via somatic mutations or from the anther walls [64], the chromosomes were analyzed by investigating the ITS region, which is related to parental inheritance [65,66,67]. The two morphological types differed from the control group and could be considered the same plant type based on their ITS regions (Figure 5 and Figure 7). Hence, these two plant types were not generated from somatic cell mutations or the anther wall. Based on the phylogenetic analysis, four (Figure 2 and Figure 3, lanes 8–11) out of seven putative haploid-derived plants (Figure 2 and Figure 3, lanes 5–11) were identified as anther-derived aneuploids (Figure 8c, Group I). The remaining three individuals (Figure 2 and Figure 3, lanes 5–7) were estimated to be diploids derived from a completely homozygous line (Figure 8c, Group II). Therefore, the aneuploid plants obtained in this study presumably underwent a mutation that occurred during meiosis.

In this study, the majority of the regenerated plants derived from anther culture were identified as heterozygous plants (228 out of 271), 33 of the remaining plants were haploid-derived homozygous diploids, two plants were haploids, and eight were identified as putative aneuploids (Figure 5 and Tables S2 and S3). Hidaka et al. [12] reported that haploid-derived diploid plants could not be obtained in sour orange. In addition, there are no reports of anther culture-derived aneuploid plants. Most anther culture-derived clementine plants were triploids, and few, or none, were haploids [30,57]. In this study, two haploid plants were obtained (although these samples were lost due to contamination), which is consistent with the results of Germanà et al. [30]. The stems of these two plants did not grow, and only small shoots proliferated as if they were stacked in layers. In some cases, the roots grew extremely thin compared with those of other plants. These results were similar to those of Chiancone et al. [57], who identified similar plant types as triploids. However, chromosomal analysis revealed that these were aneuploid plants. Therefore, estimating polyploidy based on morphological differences may cause errors. Further studies are needed to confirm whether the plants obtained in this study were triploids or aneuploids. Nevertheless, the proposed microspore culture method [68] can help obtain triploid plants more efficiently.

## 4. Materials and Methods

### 4.1. Plant Materials and Pretreatment

**Figure 9:**
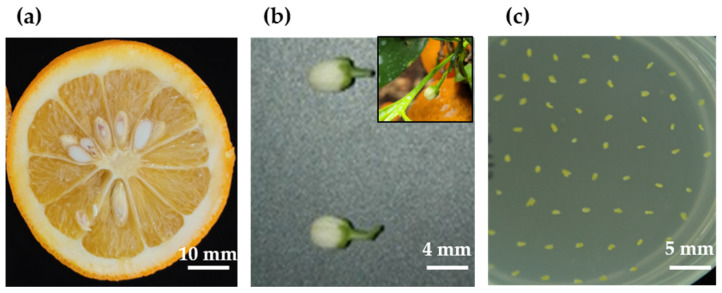
*Citrus aurantium* L. (sour orange) anther culture. (**a**) Transverse plane of a sour orange fruit; (**b**) sour orange flower bud; (**c**) anther culture in a Petri dish.

Approximately 100 sour orange (seed-forming variety) flower buds were randomly sampled from approximately 15-year-old C. aurantium L. trees obtained from the greenhouse at Citrus Research Center, RDA, Korea, in April 2020 (Figure 9a,b). The selected flower buds (3–5 mm in diameter) were stored in the dark at 4 °C for 24 h and sterilized by immersion in 70% ethanol for 30–60 s. The flower buds were then dried on sterilized filter paper for approximately 2–3 h. The petals were aseptically removed using a small pair of forceps and a scalpel, and the anthers were collected in a Petri dish (60 mm in diameter) containing a solid medium (refer to Section 4.2 for medium composition). Based on the efficiency of anther culture, N6 liquid medium supplemented with GA_3_ (1 mg·L^−1^) and spermidine (200 µM) was added—except in the control treatment—to two types of culture media [59]. The culture media were (1) N6 and (2) MS basic media supplemented with plant growth hormones (Figure 9c).

### 4.2. Composition of Anther Culture Medium and Culture Conditions

Callus cells and embryos were induced in an anther culture medium comprising N6 medium [69] supplemented with Nitsch and Nitsch vitamins [70], and MS medium [71] supplemented with MS vitamins, sucrose (50 g·L^−1^) and malt extract (500 mg·L^−1^). The following growth regulators were added to the two media: 0.2 mg·L^−1^ 2,4-dichlorophenoxy acetic acid, 0.2 mg·L^−1^ α-naphthalene acetic acid, 1.0 mg·L^−1^ kinetin, 0.8 mg·L^−1^ 6-benzyladenine, 0.43 mg·L^−1^ zeatin, and 0.44 mg·L^−1^ thidiazuron. The pH of the media was adjusted to 5.8 using 1 N KOH and 0.1 N HCl; 0.8% (*w*/*v*) agar was used for solid media. Callus and embryo induction rates were compared between anther cultures with and without N6 liquid medium supplemented with GA_3_ (1 mg·L^−1^) and spermidine (200 µM). The anthers were cultured in the dark at 4 °C for the first 14 d and then cultured under a 16 h light-dark photoperiod at 25 ± 2 °C.

### 4.3. Plant Regeneration

Callus cells induced in the anther culture medium were transferred to a somatic embryo induction medium (MS medium supplemented with 500 mg·L^−1^ malt extract and 164 mM lactose) according to the method described by Jin et al. [72] and cultured for 6 weeks. Somatic embryos were then transferred to MS medium supplemented with thidiazuron (0.5 mg·L^−1^), GA_3_ (1 mg·L^−1^), malt extract (500 mg·L^−1^), sucrose (50 g·L^−1^), and Gelrite^®^ (0.2%) to induce germination. The embryos showing shoot development were transferred and cultured in a Magenta box (PlantMedia^TM^, Dublin, OH, USA) containing MS medium with GA_3_ (0.5 mg·L^−1^), malt extract (500 mg·L^−1^), sucrose (50 g·L^−1^), and agar (8 g·L^−1^). The roots were induced following a 6-week culture period. Each of the newly formed shoots was grafted onto the cultivated citron root. Subsequently, these plants were transferred outdoors from the greenhouse for acclimation to field conditions.

### 4.4. Genetic Analysis of Regenerants

To verify whether the plants obtained from the anther culture were haploid-derived, two SSR marker primers (S. Biomedics Co., Ltd., Seoul, Korea) specific to the heterozygote were used (Table 1). Primers specific to citrus plants were used to screen 53 microsatellite markers [73]; six SSR markers specific to sour orange plants were used (Table 1). Using the donor plant as a control, total genomic DNA was extracted from approximately 0.2 g of plant material (leaves) using an automated DNA extraction system (MX 16; Promega, Madison, WI, USA). The extracted DNA was stored at −20 °C until further use. The PCR reaction consisted of genomic DNA and AccuPower^®^ Multiplex PCR PreMix (Bioneer, Corp., Daejeon, Korea), comprising 250 μM dNTPs, 1.5 mM MgCl_2_, 1 U Taq DNA polymerase, 10 mM Tris-HCl (pH 9.0), and 40 mM KCl. To this mixture, 20 ng of 0.5 μM primer was added, and the reaction volume was adjusted to 20 µL. The PCR cycle conditions were as follows: initial denaturation at 94 °C for 5 min, followed by 35 cycles of denaturation at 94 °C for 30 s, annealing at 58 °C for 30 s, and elongation at 72 °C for 40 s, followed by final extension at 72 °C for 5 min. The PCR products were then analyzed electrophoretically using the QiAxcel Advanced System (Qiagen, Hilden, Germany).

### 4.5. Analysis of Polyploidy

To verify the ploidy of the plants derived from anther culture, leaf samples were collected from each plant obtained through in vitro grafting. The samples were prepared according to Chiancone et al. [57]. The ploidy of the prepared samples was determined using flow cytometry (CyStain UV Precise PAm Flugplatz 13. 02828; Sysmex Partec GmbH, Görlitz, Germany). Approximately 0.1 g of leaf sample from heterozygous donor plants were chopped using a sharp razor blade and added to a plastic Petri dish containing 0.5 mL of nuclei extraction buffer and 2 mL of staining buffer. The samples were passed through a 30-µm Partec CellTrics^®^ filter (Sysmex Partec GmbH) directly into the sample test tube. This test tube was loaded into the machine for analysis. A total of 109 regenerant lines were analyzed.

### 4.6. Chromosomal Analysis

To confirm ploidy, the samples were pretreated and prepared according to the method described by Ha et al. [74]. Young root tips (0.5–1.0 cm) were collected from plants obtained through anther culture. The root tips were pretreated in 2 mM 8-hydroxyquinoline for 5 h at 25 °C, followed by treatment with a fixative solution (ethyl alcohol: acetic acid, 3:1). The fixed root tips were washed three times with distilled water and then treated with an enzyme mixture (0.3% cellulase, 0.3% cytohelicase, and 0.3% pectolyase) at 37 °C for 60 min. The enzyme-treated roots were then transferred to a 1.5-mL tube containing the fixative solution and homogenized by vortexing for 20 s. The homogenate was placed on ice for 5 min and then centrifuged at 13,000 rpm. The supernatant was discarded, and the pelleted material was immediately resuspended in an acetic acid-ethanol (9:1) solution. To prevent changes in chromosome morphology during chromosome preparation, the suspension was spread onto pre-warmed (80 °C) glass slides [75], which were then placed in a humid chamber and air-dried at room temperature. The slides were then counterstained with 1 µg·mL^−1^ 4,6-diamidino-2-phenylindole (Roche, Indianapolis, IN, USA), mounted in VECTASHIELD^®^ (H-1000; Vector Laboratories, Newark, CA, USA), and examined under an Olympus BX53 fluorescence microscope (100× magnification).

### 4.7. Phylogenetic Analysis

The genetic relationship between the donor plant (control plants) and anther culture-derived regenerated plants was analyzed as described previously [65,66]. The entire ITS region (ITS15.8S-ITS2) in the nuclear ribosomal DNA was analyzed by PCR using the primers ITS1F1 (5′-GAAGGATCATTGTCGACCTGCCAGCAGACG-3′) and ITS2R2 (5′-GACCTGGGGTCGCAATGCGAGCGCCGCTT-3′) [65]. The amplified PCR products were electrophoresed at 100 V for 30 min in a 1.2% agarose gel. Amplified products con-firmed on the agarose gel were extracted using the GeneAll^®^ Gel purification kit (GeneAll Biotechnology, Co., Seoul, Korea). The purified amplification products were cloned using the pLUG-Prime^®^ TA-Cloning Vector kit (iNtRON, Seongnam-si, Korea) [66]. The cloned PCR products were then sent to SolGent (SolGent Co., Daejeon, Korea) for nucleotide sequence analysis [66]; the sequences were edited using BioEdit [76] and used for phylogenetic relationship analysis via MEGA (Molecular Evolutionary Genetics Analysis) software ver. 5.2.

## 5. Conclusions

In this study, anther culture of sour orange plants was performed and haploid-derived homozygous diploid and aneuploid plants were obtained. The findings of this study can be effectively applied to mutant breeding of citrus cultivars through anther culture. More importantly, the results would be helpful in the study of anther cultures of other citrus varieties since haploid-derived homozygous diploid plants were obtained using anther culture in this study. Future research should focus on chromosomal analysis of pollination-derived individuals and incorporate microspore culture for the development of marketable, seedless varieties.

## Figures and Tables

**Table 1:** Nucleotide sequence and repeat motifs of the simple sequence repeat markers used in the study.

No.	Product Name	Primer Sequence (5′-3′)		Repeat Motif	Homozygous Cultivars [References]
		Forward	Reverse		
1	BM-CiSSR-P1	CCCCCTCTTCTTTCACACAA	GGTGAGCAGCCATCTTCTTC	(TA)6	*C. clementina* ‘Fina Sodea’, *C. erythrosa* ‘Dingjeongkyul’
2	BM-CiSSR-P2	GAATTGGGAGGACGAACTGA	CGAGCCCTAGACAGAGATGG	(AGA)7	*C. pseudogulgul*, Citrus hybrid ‘Haruka’
3	BM-CiSSR-043	ATTAGTGCGGGTAAGATGAA	AAGGATTTGGTGTAGGAAGTAA	(AAAAT)3	Woo et al. [73]
4	BM-CiSSR-226	ATTAAGGCTGGAAATGCCAC	ATTCTGCTGACGCTTCAATG	(ATT)9	Woo et al. [73]
5	BM-CiSSR-246	CCCTAGGGAAATTTGGGAAT	GCACTCGAGAGTTCTCGTTAAG	(CAT)11	Woo et al. [73]
6	BM-CiSSR-253	AATTTCCTGCTCCAAACCAG	TCCAACAACTTGAACACGGT	(TAA)14	Woo et al. [73]
7	BM-CiSSR-254	TAAAATCCCTCGGAAACAGG	CTTTGCATGTTCAACGTTCC	(ATC)6	Woo et al. [73]
8	BM-CiSSR-260	TCATCTGAACGGACCACAAT	TAACTGCACTTGCTTCCCTG	(TTC)6	Woo et al. [73]

## Data Availability

The datasets generated during and/or analyzed during the current study are available from the corresponding author on reasonable request.

## Supplementary Materials

The following supporting information can be downloaded at: https://www.mdpi.com/article/10.3390/plants11223022/s1, Table S1: Effect of media composition on callus and somatic embryo formation during anther culture of *Citrus aurantium* (sour orange); Table S2: Occurrence rate of haploid-derived plants obtained from anther culture of *Citrus aurantium* (sour orange); Table S3. Ploidy analysis of regenerated plants identified through genetic analysis; Table S4: Chromosome number of regenerated plants.
