# Supplementary material for: Anther Culture-Derived Haploids of Citrus aurantium L. (Sour Orange) and Genetic Verification of Haploid-Derived Regenerated Plants
Source: Plants (Basel). 2022 Nov 9;11(22):3022. doi: 10.3390/plants11223022 (PMC9698947; doi:10.3390/plants11223022)
Supplement: Supplementary file 1 [file plants-11-03022-s001.zip › plants-1979771-supplementary.pdf]

plants

MDPI

Table S1. Effect of media composition on callus and somatic embryo formation during anther culture of *Citrus aurantium* (sour orange)

Treatment	Number of anthers	Anthers with callus cells		Anthers with somatic embryo formation	
		n	%	n	%
N6 solid medium	100	24–26	25	2–3	2.3
N6 solid medium and/ add N6 liquid[z]	100	36–37	36	10–12	11
MS solid medium	100	24–25	25	1–2	1.3
MS solid medium/ add N6 liquid	100	34–36	35	10–12	11

[z] N6 Liquid medium supplemented with GA_3 (1 mg·L^{-1}) and spermidine (200 μM).

Table S2. Occurrence rate of haploid-derived plants obtained from anther culture of *Citrus aurantium* (sour orange)

SSR marker	Number of plants	Number of heterozygous plants[Y]		Number of haploid-derived plants			
				Number of homozygous plants		Number of putative aneuploid plants [Z]	
		n	%	n	%	n	%
CiSSR-P1	271	228	84	43	16	ND	-
CiSSR-P2	271	228	84	43	16	ND	-
CiSSR-43	271	228	84	43	16	ND	-
CiSSR-226	271	228	84	35	13	8	3
CiSSR-253	271	228	84	35	13	8	3
CiSSR-246	271	228	84	35	13	ND	-
CiSSR-254	271	228	84	35	13	8	3
CiSSR-260	271	228	84	35	13	8	3

[Y] control plants

[Z] The occurrence of hetero amplification products in a pattern different from that of the control plants. ND, Not detected.

Table S3. Ploidy analysis of regenerated plants identified through genetic analysis

Number of plants	Ploidy analysis					
	Haploid[Y]		Diploid		Putative aneuploids [Z]	
	n	%	n	%	n	%
41	2	5	33 (homozygous)	80	6	15
68	ND	-	66 (heterozygous)	97	2	3
109[X]	2	2	99	91	8	7

[Y] Two specimens were lost due to contamination

[Z] The ploidy verification pattern different from that of the diploid plants

[X] The ploidy verification pattern the number of total plants. ND, Not detected.

Table S4. Chromosome number of regenerated plants

Number of plants	Number of chromosomes							
	2n:18		2n:19		2n:20		2n:21	
	n	%	n	%	n	%	n	%
30	22	73	5	17	2	7	1	3
